# Small G-protein Rnd3/RhoE is expressed in brain cancer and affects glioma morphology, motility and invasion

**DOI:** 10.1186/2051-1426-2-S1-O4

**Published:** 2014-02-24

**Authors:** Abdelhamid Liacini, Donna Sanger, Peter Forsyth

**Affiliations:** 1 Clark Smith Integrative Brain Tumor Research Center, Department of Oncology, Clinical Neurosciences and Molecular biology, University of Calgary, and Tom Baker Cancer Centre, Calgary, Alberta, Canada; 2 Multi-Organ Transplant Center (MOTC), Histocompatibility & Immunogenetics Laboratory (HIL), King Fahad Specialist Hospital-Dammam, Saudi Arabia

## 

Malignant astrocytomas are highly invasive neoplasms infiltrating diffusely into regions of normal brain. Migration or invasion of tumor cells into normal tissue is a principal cause of mortality and is thought to be a multi-factorial process, consisting of cell interaction with ECM and adjacent cells, as well as accompanying biochemical processes supportive of active cell movement. Rho family members are known to regulate malignant transformation motility and invasion of cancer cells, but the clinicopathological significance of Rnd3 remains unclear. We evaluated the protein expression level of RhoE in gliomas tissues, cell lines and brain tumor initiating stem cells (BTSCs). Results showed that the expression of RhoE was significantly higher in patients with highly invasive gliomas. Next, we constructed a mammalian expression plasmid containing human RhoE and ectopically expressed it in U87. Ectopic expression of RhoE was found to induce membrane ruffle formation, increased migration and invasion both in vitro and in vivo and altered the relative levels of GTP-bound Rac1 and RhoA. Finally RhoE siRNA in the glioma cell line U251 inhibited cell invasion in vitro. In conclusion, the expression of RhoE appears to have a role in the pathogenesis of gliobstoma multiforme.

